# Knowledge, attitude, and practice towards breast self-examination among women: a web based community study

**DOI:** 10.3389/fpubh.2024.1450082

**Published:** 2024-10-07

**Authors:** Geetha Kandasamy, Dalia Almaghaslah, Mona Almanasef, Raseel Dhafer Abdulhadi Alamri

**Affiliations:** Department of Clinical Pharmacy, College of Pharmacy, King Khalid University, Abha, Saudi Arabia

**Keywords:** breast self-examination, knowledge, attitude, practice, women

## Abstract

**Background:**

The most common form of cancer among women is breast cancer in the Kingdom of Saudi Arabia. Thus, the purpose of this study was to evaluate women in Saudi Arabia’s Asir Region, on their knowledge, attitudes, and practices (KAP) regarding breast self-examination (BSE).

**Methods:**

The research was carried out cross-sectional and conducted from October 2023 to June 2024 in Saudi Arabia’s Asir province.

**Results:**

Out of 397 study participants, 89 (22.4%) had good knowledge, and 308 (77.6%) had poor knowledge; 185 (46.6%) had a positive attitude, and 212 (53.4%) had a negative attitude 24 (6%) had good practice 373 (94%) had poor practice about breast BSE. According to study participants, social media, 204 (51.4%), radio, television, and newspapers were the best places to learn about BSE. In this study, 316 (79.6%) had heard of BSE. 230 (57.9%) stated that breast cancer patient’s likelihood of survival increases with early detection. Only 231 (58.2%) and 247 (62.2%) agreed that breast cancer is indicated by changes in the color and shape of the breast and retraction/discharge of the breasts, respectively. Out of 147, 24 (16.3%) self-examined just one week after each menstruation. Single (OR = 6.259; 95% CI = 1.790–21.887, *p* = 0.004) and married (OR = 4.923; 95% CI = 1.509–16.056, *p* = 0.008), Single (OR = 2.736; 95% CI = 1.248–6.000, *p* = 0.012) and married (OR = 3.176; 95% CI = 1.734–5.817, *p* = 0.00) were significantly associated with good knowledge and attitude of BSE, respectively. Illiterates (OR = 0.233; 95% CI = 0.060–0.895, *p* = 0.034), pre-university (OR = 0.222; 95% CI = 0.092–0.538, *p* = 0.001), Illiterates (OR = 0.293; 95% CI = 0.114–0.755, *p* = 0.011), pre-university (OR = 0.462; 95% CI = 0.271–0.788, *p* = 0.005) are less likely to have good knowledge and attitude compared to college and university educated.

**Conclusion:**

The study revealed that few women have good knowledge, attitudes, and practices among BSE. In this study, attitude level is high in comparison to knowledge and practice. However, 94% never practiced at all. It is strongly advised that a focus be placed on improving women’s KAP regarding BSE, as well as BSE educational programs in universities and more community service activities such as health campaigns in public places.

## 1 Introduction

The most common form of cancer among women is in the Kingdom of Saudi Arabia breast cancer. Research carried out in the Kingdom has demonstrated that women’s awareness of breast cancer is inadequate. Globally, breast cancer is the most prevalent cancer that results in the greatest number of cancer-related deaths among women (1). It is a worldwide health issue affecting both modern and underdeveloped nations (2). When considering cancers of both sexes together, breast cancer ranks second overall and is quite the most common tumor-related disease in women. In 2020, it accounted for 24.5% of all cancer cases and 15.5% of cancer deaths among women globally (3). The prevalence of breast cancer in Arab nations is alarming (4). It is expected that annually, over a million women out of 17,221,671 will be diagnosed with breast cancer (5). As per the Saudi Cancer Registry (SCR) 2018 data, breast cancer accounted for 31.8% of all newly diagnosed female malignancies, placing it at the top of the list among Saudi Arabian women’s cancers (6). Breast cancer is linked to a wide range of risk factors. The primary risk factor for an increased chance of breast cancer development is being a woman.

Being over the age of 50 years, having dense breasts, having a family history of breast cancer, menstruating early and menopausal late, being pregnant late, taking birth control pills, having atypical hyperplasia of the breast, and having undergone radiation therapy in the past are the most common risk factors for breast cancer (7, 8). Women who have a first-degree relative with a family history of breast cancer are more vulnerable, and those who have already had breast cancer are more likely to get it again (9). Obesity has been shown to raise the risk of breast cancer after menopause (10). Lifestyle factors include alcohol consumption, obesity, low levels of physical activity, and diets heavy in fat and poor in fiber. Research revealed several health benefits and a decreased risk of breast cancer, including regular physical activity, maintaining a healthy weight, reducing alcohol consumption, skipping postmenopausal hormone therapy, breastfeeding, eating more fruits and vegetables, and consuming fewer animal fats (8). One non-invasive screening technique is called breast self-examination (BSE), in which a woman examines her breasts for any abnormal findings, such as masses, deformations, discharges, or inflammation, to detect them early for early treatment initiation and a higher chance of survival for breast cancer patients (11). According to American Cancer Society guidelines, women should have a mammogram every year beginning at age forty, a clinical breast examination (CBE) every three years for those in their twenties and thirties, and a yearly exam for those over forty. BSE is also advised for women starting in their twenties (12). BSE is an easy, harmless, cost-free adjuvant screening technique for early breast cancer identification. Its goal is to familiarize women as soon as possible with the feel and look of their breasts (13). How breast cancer screening programs are implemented is heavily influenced by several elements such as healthcare organizations, national policies, and resource availability. It will be simpler to accomplish this common objective with the cooperation and knowledge of numerous healthcare specialists. Thus, the purpose of this study was to evaluate women in Saudi Arabia’s Asir Region, on their knowledge, attitudes, and practices (KAP) regarding BSE.

## 2 Methods

### 2.1 Study design

The study was a cross-sectional, self-administered, anonymous web survey conducted in the Asir province of Saudi Arabia between October 2023 and June 2024.

### 2.2 Study population/criteria

This study recruited women over the age of 20 in different regions of Asir, Saudi Arabia. Participants with known psychiatric problem and already been diagnosed to have breast carcinoma were excluded. It was made clear to each participant that taking part in the study is completely optional. Participants who submitted questionnaires that were incomplete or did not give their consent to participate in the study were not included.

### 2.3 Sampling procedure and sample size

Using a non-probability convenience sampling method, study participants were selected. The sample size was determined using the Raosoft online sample size calculator, based on the number of females residing in the Asir region between the ages of 20 and 70 years, which is 600,103. With a response distribution set at 50%, a margin of error at 5%, and a confidence level of 95%, the minimum sample size required to minimize inaccuracies and enhance the study’s reliability was 384. A total of 423 questionnaires were returned, of which 26 were incomplete and thus eliminated. Therefore, 397 questionnaires were included in the study.

### 2.4 Data collection instruments

A specially created, validated, self-administered questionnaire was adopted from previous study (14–16). Part 1 consists of socio-demographic characteristics such as age, marital status, women’s level of education, occupation, husband’s level of education, family history of breast cancer. Part 2 consists of knowledge (*n* = 8), part 3 consists of attitude (*n* = 11), and part 4 consists of practice (*n* = 6) items and last section contains the sources of information. The total scores for Knowledge, Attitude, and Practice were calculated separately. Each domain was then categorized into good or poor levels based on a 70% cut-off point, which was applied to the maximum possible score within each specific domain (Knowledge, Attitude, and Practice) (17).

The Cronbach’s alpha test for the questionnaire’s general KAP was 0.86, 0.84 and 0.73. A week following each menstrual cycle, at least once a month, one should check or perform a BSE. This is considered good practice. When someone practices BSE at a period other than the proper cycle, it is considered poor practice (18). Meanwhile, categorical variables were expressed by the frequencies and percentage. The total scores for the KAP was calculated and then was categorized into good knowledge or poor knowledge, good attitude or poor attitude, good practice or poor practice.

### 2.5 Data analysis

The collected data were pre-coded and analyzed using Statistical Package for social sciences version 20.0 (SPSS). Descriptive statistics like percentage and number were used to characterize continuous variables. The significant differences between the independent factors and BSE KAP were assessed using multivariate logistic regression analysis. *p* < 0.05 was regarded as significant.

### 2.6 Ethics approval

The institutional review committee at King Khalid University ECM ≠ 2023–3,104 granted ethical approval. Before any data was collected, each participant’s written consent was also obtained.

## 3 Results

Of the 397 individuals who completed the survey, 139 (or 35%) fell within the 20–29 age range. There were 97 (24.4%) people in the 30–39 age range, 112 (28.1%) in the 40–49 age range, and 49 (12.3%) in the 50–70 age range. Of the participants, 176 (44.3%) were married, 125 (31.5%) were single, and 96 (24.2%) were divorced or widowed. The percentage of illiterate women was 34 (86%), followed by pre-university (153; 38.5%), and college and university (210; 52.9%). As per the occupation, 125 (31.5%) were students, 193 (48.6%) were employees, and 79 (19.9%) were housewives. Husbands level of education 133 (33.5%) has studied in college and university, 16 (4%) were illiterate, 114 (28.7%) were pre-university. 44 (11.1%) had a history of breast cancer while 353 (88.9%) affirmed they did not (Table 1).

**Table 1:** Sociodemographic characteristics of study population.

Variables	Category	Number (%)
Age	20–29 Years	139 (35%)
	30–39 Years	97 (24.4%)
	40–49 Years	112 (28.2%)
	50–70 Years	49 (12.3%)
Marital Status	Single	125 (31.5%)
	Married	176 (44.3%)
	Divorced/Widowed	96 (24.2%)
Women’s level of education	Illiterate	34 (86%)
	Pre-university	153 (38.5)
	College and university	210 (52.9)
Occupation	Housewife	79 (19.9%)
	Employee	193 (48.6%)
	Student	125 (31.5%)
Husband’s level of education	Illiterate	16 (4%)
	Pre-university	114 (28.7%)
	College and university	133 (33.5)
	NA	134 (33.8)
Family history of breast cancer	Yes	44 (11.1%)
	No	353 (88.9%)

### 3.1 Knowledge toward breast self-examination among women

Out of 397 study participants, 89 (22.4%) had good knowledge, 3.8 (77.6%) had poor knowledge about breast self-examination (BSE) (Table 2). In this study, 316 participants (79.6%) had heard of BSE, while 81 (20.4%) had not. About 58% believed early detection improves breast cancer survival, and 58.2% thought that changes in breast color and shape indicate breast cancer. Nearly 49% agreed that lumps in the breast and under the armpit are symptoms of breast cancer, and 62.2% identified retraction and discharge of the breasts as indicators. Most (55.9%) thought that only females should perform BSE, and 39.3% were unsure how often BSE should be done. Details were provided in Table 3.

**Table 2:** Scores of knowledge, attitude, and practice about breast self-examination among women.

	Knowledge number (%)	Attitude number (%)	Practice number (%)
Good	89 (22.4%)	185 (46.6%)	24 (6%)
Poor	308 (77.6%)	212 (53.4%)	373 (94%)

**Table 3:** Knowledge toward breast self-examination among women.

Questions	Responses	Number (%)
Have you ever heard of breast self-examination before?	Yes	316 (79.6%)
	No	81 (20.4)
Does a breast cancer patient’s likelihood of survival increase with early detection?	Agree	230 (57.9%)
	Disagree	167 (42.1%)
Breast cancer is indicated by changes in the color and shape of the breast.	Agree	231 (58.2%)
	Disagree	166 (41.8%)
Breast cancer symptoms include lumps in the breast and under the armpit.	Agree	194 (48.9%)
	Disagree	203 (51.1%)
Breast cancer is indicated by the retraction and discharge of the breasts.	Agree	247 (62.2%)
	Disagree	150 (37.8%)
Who is the appropriate person to do a breast self-examination?	Males only	4 (1%)
	Females only	222 (55.9%)
	Both	106 (26.7%)
	Do not know	65 (16.4%)
Is breast self-examination an important component of early breast cancer detection?	Important	103 (25.9%)
	Not important	124 (31.2%)
	I do not know	170 (42.8%)
How frequently should one undertake a breast self-examination?	Weekly	13 (3.3%)
	Monthly	117 (29.5%)
	Yearly	111 (28%)
	Do not know	156 (39.3%)

Logistic regression (LR) is usually applied to find out the probability of a particular outcome about several other independent variables. Here, LR was used to find out the probability (Odds) of the students having a good knowledge level regarding BSE compared to that of students having poor knowledge. That is, among the independent variables, the regression results show that marital status and women’s level of education have significant effects on good knowledge levels. The details were provided in Table 4.

**Table 4:** Multiple logistic regression analysis for association of variables and knowledge.

Independent variables	Groups	B value	*P* value	Odds Ratio (OR)	95% Confidence interval (CI)	
Intercept		−2.618	0.012		Lower	Higher
Age	20–29 Years	0.443	0.462	1.557	0.478	5.071
	30–39 Years	−0.887	0.138	0.412	0.128	1.331
	40–49 Years	−0.708	0.219	0.493	0.159	1.523
	50–70 Years					
Marital Status	Single	1.834	0.004	6.259	1.790	21.887
	Married	1.594	0.008	4.923	1.509	16.056
	Divorced/Widowed					
Women’s Level of education	Illiterate	−1.459	0.034	0.233	0.060	0.895
	Pre-university	−1.504	0.001	0.222	0.092	0.538
	College and university					
Occupation	Housewife	−0.431	0.439	0.650	0.218	1.937
	Private employee/Government employee	−0.330	0.498	0.719	0.277	1.865
	Student					
Husband’s level of education	Illiterate	−1.717	0.121	0.180	0.020	1.577
	Pre-university	−0.033	0.952	0.968	0.335	2.793
	College and university	0.736	0.060	2.087	0.971	4.487
	N.A					
Family history of breast cancer	Yes	0.537	0.438	1.711	0.441	6.636
	No					

### 3.2 Attitude toward breast self-examination among women

In this study, 185 participants (46.6%) had a positive attitude toward BSE, while 212 (53.4%) had a negative attitude. Most women (60.7%) agreed that all women should conduct BSE, and 78.8% considered self-examination essential. Over half of the women (54.2%) expressed concern for their breasts, while 56.2% preferred seeking medical care if a lump was found. Only 43.3% felt that BSE was not embarrassing, and 58.7% believed BSE is not a waste of time. After performing BSE, 57.2% of the women were satisfied. While 44.3% felt uncomfortable with monthly self-examination, 55.7% felt comfortable. About 67.3% thought BSE could be beneficial, and 51.6% were consistently concerned about breast cancer. The details were provided in Table 5.

**Table 5:** Attitude toward breast self-examination among women.

Questions	Responses	Number (%)
It is recommended that all women perform a Breast self examination.	Agree	241 (60.7%)
	Disagree	156 (39.3%)
It is essential to self-examine your breasts	Agree	313 (78.8%)
	Disagree	84 (21.2%)
I care about my breasts	Agree	215 (54.2%)
	Disagree	182 (45.8%)
My preference is to seek treatment from a medical facility if there is a lump.	Agree	223 (56.2%)
	Disagree	174 (43.8%)
I find nothing embarrassing about BSE	Agree	172 (43.3%)
	Disagree	225 (56.7%)
It is not time-wasting to perform a breast self-examination.	Agree	233 (58.7%)
	Disagree	164 (41.3%)
I feel satisfied after completing a breast self-examination.	Agree	227 (57.2%)
	Disagree	170 (42.8%)
I feel comfortable doing a breast self-examination once a month.	Agree	221 (55.7%)
	Disagree	176 (44.3%)
Do you believe that doing a breast self-examination can be beneficial?	Agree	267 (67.3%)
	Disagree	130 (32.7%)
I would like to undertake a Breast Self Examination since I always worry that I may have breast cancer.	Agree	205 (51.6%)
	Disagree	192 (48.4%)

Logistic regression was applied. That is, among the independent variables, the regression results show that marital status and women’s level of education have significant effects on good attitude levels. Details were provided in Table 6.

**Table 6:** Multiple logistic regression analysis for the association of variables and attitude.

Independent variables	Groups	*B* value	*p* value	Odds ratio (OR)	95% Confidence interval (CI)	
Intercept		−0.401	0.531		Lower	Higher
Age	20–29 Years	−0.049	0.912	0.952	0.398	2.277
	30–39 Years	−0.425	0.291	0.654	0.297	1.439
	40–49 Years	−0.650	0.101	0.522	0.240	1.134
	50–70 Years					
Marital status	Single	1.007	0.012	2.736	1.248	6.000
	Married	1.156	0.000	3.176	1.734	5.817
	Divorced/Widowed					
Women’s level of education	Illiterate	−1.227	0.011	0.293	0.114	0.755
	Pre-university	−0.773	0.005	0.462	0.271	0.788
	College and university					
Occupation	Housewife	−0.725	0.078	0.484	0.217	1.084
	Private employee/Government employee	−0.211	0.553	0.810	0.404	1.624
	Student					
Husband’s level of education	Illiterate	−0.209	0.745	0.812	0.231	2.852
	Pre-university	0.378	0.287	1.459	0.728	2.924
	College and university	0.712	0.026	2.038	1.091	3.808
	N.A					
Family history of breast cancer	Yes	0.012	0.974	1.012	0.495	2.070
	No					

### 3.3 Practice toward breast self-examination among women

Out of 397 participants, 24 (6%) had good BSE practices, while 373 (94%) had poor practices. Of those who self-examined, 16.3% did so one week after menstruation, 74.1% at any time during the cycle, and 9.5% before menstruation. Among those who did not self-examine, 58% felt it was unnecessary, and 42% did not know how. Regarding technique, 42.6% used their palms and three fingers, while 48.9% were unsure. For locations, 21.7% performed BSE lying on the bed, and 49.1% did not perform BSE. Of the ladies, 9.6% would inform their mother, 14.4% their spouse, and 25.2% would remain quiet out of embarrassment. Most (69.3%) preferred to perform BSE themselves, while 30.7% preferred a nurse or doctor. Details were provided in Table 7. For information sources, 51.4% relied on media.

**Table 7:** Practice toward breast self-examination among women.

Questions	Responses	Number (%)
Have you ever self-examined your breasts?	Yes	147 (37%)
	No	250 (63%)
If ‘Yes’ when did you perform it?	Just a week after each menses	24 (16.3%)
	Anytime during menses	109 (74.1%)
	Before menses	14 (9.5%)
If the answer is “no,” then why have not you done it?	Fear of detecting an abnormality	143 (36.02%)
	Not necessary	76 (19.14%)
	Do not know how to self-examine	105 (26.44%)
	Too busy	73 (18.38%)
How does one perform a breast self-examination?	Palpate with one finger	34 (8.6%)
	Palpate with palm and three finger	169 (42.6%)
	I do not know	194 (48.9%)
Where do you typically examine your breasts?	In front of the mirror	56 (14.1%)
	Lying on the bed	86 (21.7%)
	In the bathroom	60 (15.1%)
	Did not do at all	195 (49.1%)
How would you respond if you noticed something unusual about your breasts?	Tell mother	38 (9.6%)
	Tell spouse	57 (14.4%)
	Consult doctor/nurse	158 (39.8%)
	Not do anything about it due to embarrassment	100 (25.2%)
	Option for traditional healing	44 (11.1%)
Which method of doing a breast exam do you prefer?	By nurse/doctor	122 (30.7%)
	By yourself	275 (69.3%)

Among the independent variables, the regression results show that age, marital status and women’s level of education, occupation, husband level of education, family history of breast cancer have no significant effects on good practice levels (*p* > 0.05). Details were provided in Table 8.

**Table 8:** Multiple logistic regression analysis for association of variables and practice.

Independent variables	Groups	B value	*p* value	Odds ratio (OR)	95% Confidence interval (CI)	
Intercept		−2.782	0.050			
Age	20–29 Years	0.608	0.610	1.837	0.177	19.037
	30–39 Years	0.634	0.586	1.885	0.193	18.444
	40–49 Years	1.045	0.346	2.843	0.324	24.965
	50–70 Years					
Marital status	Single	0.384	0.609	1.469	0.337	6.401
	Married	−0.070	0.915	0.933	0.262	3.325
	Divorced/Widowed					
Women’s level of education	Illiterate	0.331	0.709	1.392	0.244	7.930
	Pre-university	0.217	0.712	1.243	0.391	3.946
	College and university					
Occupation	Housewife	−0.889	0.274	0.411	0.084	2.021
	Private employee/Government employee	−0.760	0.258	0.468	0.125	1.745
	Student					
Husband’s level of education	Illiterate	0.096	0.932	1.101	0.123	9.819
	Pre-university	−0.682	0.383	0.505	0.109	2.342
	College and university	0.181	0.737	1.199	0.417	3.449
	N.A					
Family history of breast cancer	Yes	−0.402	0.560	0.669	0.173	2.585
	No					

## 4 Discussion

The purpose of this study was to evaluate knowledge, attitudes, and practices (KAP) regarding BSE among women in Asir Region, Saudi Arabia. We found that most of our respondents were married 44% followed by single 31.5% Saudi women. More than half of the women were degree holder and the majority were employees. One third of participants husband level of education also degree holders. A small percentage of participants indicated a family history of breast cancer. Among the participants, a certain proportion had good knowledge about breast self-examination (BSE), while the majority had poor knowledge. A notable portion of participants held a positive attitude toward BSE, whereas a larger proportion had a negative attitude. Additionally, a small fraction of participants practiced BSE well, whereas the majority had poor practice habits.

Among the three most common signs of breast cancer, breast lump, nipple discharge and retraction, and changes in breast color and shape were reported by over half of the respondents. This is consistent with three earlier research findings (19–21). Alsareii et al. conducted a study in Saudi Arabia revealed that participants had strong knowledge of BC inadequate knowledge of its symptoms (21). A lack of basic knowledge about breast cancer and a fear of practicing BSE are evident (22). According to our research, participants knew about breast cancer. Other significant features, such as breast cancer symptoms and risk factors linked to the disease’s progression, are, still little understood. Surprisingly, even though the majority of participants had bachelor’s degrees and were highly educated, 77.6% of them had poor knowledge. However, only 6% of women actually performed breast self-examination (BSE), with the majority of women (80%) having heard of the practice due to a fear of finding an abnormality.

This result is consistent with Jobran et al. (23), in contrast to our study one third of their population practice BSE (24). Comparable findings were noted in other research; participants in Malaysia and Nigeria had inadequate knowledge with 82.3 and 86.2%, respectively (25, 26). Our results indicate a positive correlation between education level and BSE knowledge, with higher education (college and university) levels being linked to greater disease awareness. This correlation has also been observed in other studies (25–27). A positive link has been discovered between BSE and education level. Higher educated women are more likely to be knowledgeable of BSE. Findings from reviews of the previous studies are consistent (28, 29). Higher educated women’s perceptions of health and their attitudes about breast cancer have been found to be positively correlated (30). In our study, it was discovered that married women engaged in higher BSE activity when the marital status variable with BSE was assessed using the LR model. Research studies also highlight the fact that married women undertake BSE more frequently (31, 32). It is also believed that women who have higher education levels are more knowledgeable and aware of breast cancer.

When it came to knowledge and practice, this study’s result showed 46.6% of participants had a favorable attitude toward breast self-examination is similar to an Ethiopian study that found 46.3% of participants in Adwa town (33). The majority of participants in our study believed that self-examination of the breasts is crucial, and that it is that all women conduct a BSE. In two Yemeni studies conducted in 2016 and 2019, women who visited primary healthcare centers showed a range of knowledge from inadequate to satisfactory (34, 35). The curriculum’s inclusion of an illustrated BSE guide contributed to the high degree of BSE practice among Alexandria University’s nursing students (36). Education initiatives also made a significant contribution to improving knowledge and identifying breast symptoms (37). The percentage of women in Gaza that conduct BSE is low; 40% have never done so, and 76.7% of participants are aware of the practice (38).

The majority of participants in our study (94%) reported poor practice as their most frequent outcome. Furthermore, a study carried out in Iran found that 74.8% of women never practice BSE and that 9.8% of women performed their first breast examination after experiencing breast pain (26). A study conducted in Malaysia also found that 60.4% of the community, were ignorant about BSE. Furthermore, a study conducted by Nde et al. found that while less than 3% of female university students frequently exercised BSE on a monthly basis, only 30% of them had done so at least once (27). Therefore, all of the above investigations concluded that bad practices exist generally, while there is considerable difference among populations because of varying degrees of education and understanding of breast cancer.

Of the participants in this study, 204 (51.4%) stated that social media, newspapers, radio, and television were the best sources of information regarding BSE. A prior survey found that the majority of women (57%) who knew anything about breast cancer got their information from the media (television, radio, and newspapers). Additional sources included hospital employees (19%), friends and family (11%) (23). Younger participants with post-secondary education at the University of Buea in Cameroon (27) regarded television as their primary information source. The majority of participants in our study were aware of breast cancer, and social media was their primary source of information, which is similar to the findings of the Alshahrani study (39). Few participants in another study obtained some information about BSE via the media; the majority of their information was gathered from breast cancer campaigns.

## 5 Limitations

There are several restrictions on this study. As the study used a self-reported online questionnaire and non-probability sampling, which can introduce recollection biases, the results may not be generalizable.

## 6 Recommendations

In the future, we will investigate the use of dedicated cell phone apps (40) (such as BreastTest, Igyno, and Breast Cancer Indicators) and e-learning programs (41), which can help improve women’s knowledge and performance in BSE. These methods begin to be used worldwide, though still with insufficient diffusion (42).

## 7 Conclusion

The study revealed that very few women had good knowledge, attitudes, and practices among BSE. In this study, attitude level is high in comparison to knowledge and practice. However 94% never practiced at all. It is strongly advised that a focus be placed on improving women’s KAP regarding BSE, as well as on reinforcing the BSE educational programs in universities and more community service activities such as health campaigns in public places.

## Data Availability

The original contributions presented in the study are included in the article/supplementary material, further inquiries can be directed to the corresponding author.
